# Loss of legumain induces premature senescence and mediates aging‐related renal fibrosis

**DOI:** 10.1111/acel.13574

**Published:** 2022-02-23

**Authors:** Dekun Wang, Lichun Kang, Chuan'ai Chen, Jiasen Guo, Lingfang Du, Donghui Zhou, Gang Li, Yuying Zhang, Xue Mi, Mianzhi Zhang, Shuxia Liu, Xiaoyue Tan

**Affiliations:** ^1^ Department of Pathology School of Medicine Nankai University Tianjin China; ^2^ College of Life Science Nankai University Tianjin China; ^3^ Nephrology Division The Second Hospital of Tianjin Medical University Tianjin China; ^4^ Dongfang Hospital of Beijing University of Chinese medicine Beijing China; ^5^ Hebei Key Laboratory of Nephrology Department of Pathology Hebei Medical University Shijiazhuang China

**Keywords:** aging‐related renal fibrosis, autophagy, legumain (asparagine endopetidase), premature senescence

## Abstract

Aging is an independent risk factor for acute kidney injury and subsequent chronic kidney diseases, while the underlying mechanism is still elusive. Here, we found that renal tubules highly express a conserved lysosomal endopeptidase, legumain, which is significantly downregulated with the growing of age. Tubule‐specific legumain‐knockout mice exhibit spontaneous renal interstitial fibrosis from the 3rd month. In the tubule‐specific legumain‐knockout mice and the cultured legumain‐knockdown HK‐2 cells, legumain deficiency induces the activation of tubular senescence and thus increases the secretion of profibrotic senescence‐associated cytokines, which in turn accelerates the activation of fibroblasts. Blockage of senescence mitigates the fibrotic lesion caused by legumain deficiency. Mechanistically, we found that silencing down of legumain leads to the elevated lysosome pH value, enlargement of lysosome size, and increase of lysosomal voltage dependent membrane channel proteins. Either legumain downregulation or aging alone induces the activation of nuclear transcription factors EB (TFEB) while it fails to further upregulate in the elderly legumain‐knockdown tubules, accompanied with impaired mitophagy and increased mitochondrial ROS (mtROS) accumulation. Therapeutically, supplementation of exosomal legumain ameliorated fibronectin and collagen I production in an *in vitro* coculture system of tubular cells and fibroblasts. Altogether, our data demonstrate that loss of legumain in combined with aging dysregulates lysosomal homeostasis, although either aging or legumain deficiency alone induces lysosome adaptation via stimulating lysosomal biogenesis. Consequently, impaired mitophagy leads to mtROS accumulation and therefore activates tubular senescence and boosts the interstitial fibrosis.

## Graphical abstract

The underlying mechanism on the aging‐related renal fibrotic injury is still elusive. Our study here demonstrates that loss of legumain in combined with aging dysregulates lysosomal homeostasis. Consequently, impaired mitophagy leads to mtROS accumulation and therefore activates tubular senescence and boosts the interstitial fibrosis.

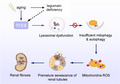

## 1 INTRODUCTION

Aging is an independent risk factor for acute kidney injury and its consequent chronic kidney disease (CKD). During aging or CKD progress regardless of cause, renal interstitial fibrosis is not only major pathological manifestation but also pathogenic factor (O'Sullivan et al., 2017). Many analogies can be drawn between aging and CKD, which implies that understanding aging‐related fibrosis is a constructive approach to decipher effective treatments for CKD (Ruiz‐Ortega et al., 2020).

As the major cell population in the kidney, renal tubules play a central role in the fibrotic response (Bonventre, 2014). Cell cycle arrest at G2/M in proximal tubule cells after kidney injury results in the abnormal amplification of profibrogenic factors (Yang et al., 2010). Cellular senescence is a permanent cell cycle arrest induced by diverse stressors including shortening of telomeres, oncogenes, DNA damage, and mechanical stress (Childs et al., 2015). Compelling evidences support that aspects of the senescence program are active during either renal aging or diverse disease conditions (Sturmlechner et al., 2017). Chronic senescence is thought to be deleterious and usually related to phenotypes of aging (Baker et al., 2011; Wolstein et al., 2010). Most recently, Mylonas et al verified the determinant role of senescent renal epithelia in fibrosis and regenerative capacity after renal injury. Targeting senescent cells, therefore, represent a potential treatment to protect aging and vulnerable kidneys (Mylonas et al., 2021). It is currently accepted that distinct phenotypic traits of senescent cells and the genomic profile that encodes the so‐called “senescence‐associated secreted phenotype (SASP)” act as determinants of senescent outcomes (Tchkonia et al., 2013).

Autophagy is a lysosome‐dependent, self‐clearance process responsible for degradation of cytoplasmic components (Klionsky, 2007). Renal tubules reabsorb most filtered solutes in a highly energy‐consuming process and proliferation is rare in renal tubules under physiological conditions compared with other epithelial cells with an active transport function (Tojo, 2013; Yamamoto et al., 2017). Considering the combination of long cellular life and high protein load, it is logical to reason that well‐being of tubular cells highly depends on adequate capacity of lysosomal adaptation. It has been reported that renal tubules of elderly mice have a higher basal autophagic flux compared with young mice, and elderly kidneys are more reliant on autophagy for removal of damaged proteins and organelles (Yamamoto et al., 2016). Dysfunctional lysosomes hinder autophagy, which results in accumulation of dysfunctional organelles, protein aggregates, and toxic reactive oxygen species (ROS) (Festa et al., 2018; Isaka et al., 2011). To date, the regulatory mechanism of the autophagy‐lysosome response in elderly tubules remains uncharacterized.

As a highly conserved lysosomal endopeptidase, legumain belongs to a family of cysteine proteases and shows strict specificity for hydrolysis of asparaginyl bonds (Chen et al., 1997). The specific cleavage functions of legumain have been implicated in various contexts including antigen processing in dendritic cells and the pathogenesis of neurofibrillary diseases via its site‐specific cleavage of toll‐like receptors and tau (Maschalidi et al., 2012; Sepulveda et al., 2009; Zhang et al., 2014, 2017). Expression of legumain in kidney proximal tubules is particularly abundant and mice lacking legumain develop automatous renal interstitial fibrosis (Miller et al., 2011). Our previous data showed that deletion of legumain aggravates renal fibrosis in the mouse model of unilateral ureteral obstruction (Wang et al., 2018). Although it is undefined whether this effect is related to the pro‐aging feature triggered by legumain deficiency, these data indicate an intrinsic correlation between this unusual lysosomal protease and the pathogenesis of kidney fibrotic lesions.

Here, we demonstrate that loss of legumain occurs with aging in renal proximal tubules. Depletion of legumain accelerates premature senescence and promotes aging‐related renal fibrosis. In the elderly kidney, loss of legumain disequilibrates the autophagy‐lysosome balance, which impairs mitophagy and leads to accumulation of mitochondrial ROS (mtROS). In terms of application, supplementation of exosomal legumain demonstrates potential on alleviating premature senescence and aging‐related renal fibrosis.

## 2 MATERIALS AND METHODS

### 2.1 Animal models

To obtain renal proximal tubule‐specific legumain‐knockout (*Lgmn* ^△Tub^) mice, we purchased *Lgmn^loxp^* ^/^ *^loxp^* mice and γ‐GT‐Cre^+/+^mice from Cyagen Biosciences Inc (Suzhou, China). Mice were on a pure C57/BL6J background. We bred *Lgmn^loxp^* ^/^ *^loxp^* mice with γ‐GT‐Cre^+/+^ mice, and wild‐type (*Lgmn^loxp^* ^/^ *^loxp^* × γ‐GT‐Cre^−/−^) mice were used as control (*Lgmn* ^WT^). Breeding and genotyping were done according to standard procedures. Male *Lgmn* ^△Tub^ mice aged 1, 3, 6, 10, and 18 months were used in these experiments.

To obtain CAG‐RFP‐EGFP‐LC3 labeled *Lgmn* ^WT^ and *Lgmn* ^KO^ mice, conventional legumain‐knockout (*Lgmn* ^KO^) mice were established using homologous recombination methods and generated by Cyagen Biosciences Inc. *Lgmn^KO^* mice were mate with CAG‐RFP‐EGFP‐LC3 transgenic mice (The Jackson Laboratory, #027139). Mice aged 3 and 18 months were used for further studies. All mice used in this study were on a pure C57/BL6J background and were housed with controlled temperature (22℃), humidity, and lighting. Mice were used under the University of Nankai's Institutional Animal Use and Care Committee's approval.

### 2.2 Human kidney specimens

Kidney biopsies were obtained from the Second Hospital of Tianjin Medical University (Tianjin, China). Samples analyzed were normal para‐carcinoma tissue of renal carcinoma patients aged from 35 to 86 years old (*n* = 16). Informed consents were obtained from all participants of this study. Briefly, fresh tissues were separated for two parts. One part of the samples was fixed in 4% paraformaldehyde for histological studies. The other part was directly store at −80℃ for RNA and protein extraction use. The information of patients is listed in Table S1. All studies involving human kidney sections were performed in accordance with the Chinese Ministry of Health national guidelines for biomedical research and approved by the Human Research Ethics Committee of Nankai University.

### 2.3 Isolation of murine renal tubules and cell culture

Mouse primary tubular epithelial cells (PTECs) were isolated and cultivated following a modified previously established protocol (Luo et al., 2018). Briefly, kidneys were collected and the medullary regions were removed, then minced into 4–6 slices. Kidney slices were transferred in reaction tube containing 1 mg/ml collagenase type II in incubation solution (Sigma‐Aldrich, St. Louis, MO) for 1 h at 37°C. After neutralization with FBS, the tissues were filtered through 40‐ and 70‐μm strainers sequentially and then centrifuged to collect the tubules.

Isolated tubules were suspended in PTECs culture medium. Cells were grown in cell culture dishes for 3–7 days to reach 60%–80% confluence. PTECs culture medium was DMEM/F12 supplemented with 10% FBS, 1% P/S, 5 mg/ml insulin (Sigma‐Aldrich), 25 ng/ml epidermal growth factor (Sigma‐Aldrich), and 5 mg/ml transferrin (Sigma‐Aldrich).

Human proximal cell line HK‐2 was cultured in DMEM/F12 supplemented with 10% fetal bovine serum (FBS; Biological Industries, Israel) and 1% penicillin and streptomycin (P/S; Thermo Fisher Scientific, Waltham, MA). Human foreskin fibroblast cell line BJ was cultured in MEM supplemented with 10% FBS, 1% P/S, and 1% NEAA (Thermo Fisher Scientific). Mouse fibroblast cell line NIH‐3T3 was cultured in high glucose DMEM supplemented with 10% FBS and 1% P/S. The cell lines were obtained from ATCC (Manassas, VA). Mouse primary tubular epithelial cells (PTECs) were isolated and cultivated as described previously. Briefly, kidneys collected were minced into small pieces, and then digested in 1 mg/ml collagenase (Sigma‐Aldrich, St. Louis, MO) for 1 h at 37°C. After neutralization with FBS, the tissues were filtered through 40‐ and 70‐μm strainers sequentially and then centrifuged to collect the tubules. Tubules were suspended in PTECs culture medium. Cells were grown in cell culture dishes for 3–7 days to reach 60%–80% confluence. PTECs culture medium was DMEM/F12 supplemented with 10% FBS, 1% P/S, 5 mg/ml insulin (Sigma‐Aldrich), 25 ng/ml epidermal growth factor (Sigma‐Aldrich), and 5 mg/ml transferrin (Sigma‐Aldrich).

### 2.4 Western blotting

Kidney tissue samples and cell pellets were homogenized in RIPA buffer that contains protease and phosphatase inhibitor cocktails (Sigma‐Aldrich). The protein concentration was determined by the Bradford Assay (Thermo Fisher Scientific). Equal amounts of proteins were separated on a 10%–15% SDS‐PAGE gel and were then transferred to a PVDF membrane (EMD Millipore, Billerica, MA). After blocking with 5% dry skim milk, the membranes were incubated overnight at 4°C with primary antibodies. The following primary antibodies were used: legumain, fibronectin, α‐SMA, CTGF, PAI‐1, p21, LAMP1, and β‐actin (Santa Cruz Biotechnology, Dallas, Texas, USA); collagen I, TGFβ1, ATP6V0D1, ATP6V1G1, p62/SQSTM1, prohibitin, and Pink1 (Abcam, Cambridge, MA).

p16^Ink4a^, Tomm20, and Parkin (Proteintech, Rosemont, IL); LC3 (MBL Life Science, Tokyo, Japan), and TFEB (Cell Signaling Technology, Danvers, MA). After washing with TBST buffer three times, the membranes were probed with respective HRP‐conjugated secondary antibodies (Santa Cruz Biotechnology) for 1 h at room temperature and then visualized using an ECL kit (EMD Millipore).

### 2.5 Sirius red staining

Kidney tissues were fixed in 4% paraformaldehyde, dehydrated through a graded series of ethanol solutions, embedded in paraffin, and sectioned at 5 μm thicknesses. Sirius red staining was performed using a Picrosirius Red Stain Kit (Abcam), following the manufacturers’ protocol. Kidney sections were deparaffinized, washed, and then sequentially stained with picric acid and Sirius red solutions. Sections were washed, dehydrated, and then mounted with neutral resin. Quantification of the Sirius red‐positive area was performed in random cortical images (×400, 10 fields/kidney) by counting the percentage of positively stained areas in each microscopic field.

### 2.6 SA‐β‐Gal activity assay

SA‐β‐gal staining was performed in accordance with the manufacturer's instructions (Beyotime, Shanghai, China). Briefly, frozen kidney tissues (5 μm thick) or cells were fixed in 4% paraformaldehyde for 15 min and then washed in PBS for 3 times. Then, the fixed sections or cells were incubated with reaction buffer overnight at 37°C without CO_2_. The sections were counterstained with eosin for 5 min and then dehydrated. Quantification of the SA‐β‐gal‐positive area was performed in random cortical images (×400, 10 fields) by counting the percentage of positively stained areas in each microscopic field.

### 2.7 Immunohistochemistry and Immunofluorescence Staining

For IHC analysis, 5‐μm‐thick kidney sections were used. After deparaffinization and rehydration, antigen retrieval was performed using citrate buffer (0.1 M citric acid and 0.1 M sodium citrate, pH 6.4). The sections were blocked with 5% goat serum and then incubated with primary antibodies overnight at 4°C. Sections were incubated with biotinylated anti‐rabbit or mouse secondary antibodies (Vector Laboratories, Burlingame, CA) for 2 h at room temperature and then incubated with streptavidin‐peroxidase complex for 1 h at room temperature. Antibody signals were detected using a DAB kit (Vector Laboratories). Sections were counterstained with hematoxylin and then dehydrated. Quantification of the antigen‐positive area was performed in random cortical images (×400, 10 fields/kidney) by counting the percentage of positively stained areas in each microscopic field.

For IF staining, shCtrl/shLGMN HK‐2 cells, and *Lgmn* ^WT^ and *Lgmn* ^KO^ PTECs cultured on glass‐bottom dishes (Thermo Fisher) were washed with PBS and then fixed in 4% paraformaldehyde for 15 min. After washing, the fixed cells were blocked with 5% goat serum and then incubated with primary antibodies overnight at 4°C. Then, cells were incubated with Alexa Fluor^TM^ 594 anti‐rabbit secondary antibody (Invitrogen, Thermo Fisher Scientific) for 1 h at room temperature, washed, dried, and then mounted with mounting medium with DAPI (Vector Laboratories). The cells were imaged under a confocal microscope (model FV1000, Olympus).

### 2.8 Hydroxyproline colorimetric assay

Hydroxyproline assay was performed according to the manufacturer's instructions (Biovision, Milpitas, CA). Kidney samples were prepared for an equal weight and then hydrolyzed in 6N HCl at 120°C for 3 h. Samples were reacted with the Chloramine T at room temperature for 5 min, then the products were incubated with DMAB reagent for 90 min, at 60°C. Absorbance at 560 nm was measured using Clariostar microplate reader (BMG Labtech, Offenburg, Germany).

### 2.9 Real‐time polymerase chain reaction

Kidney tissues and cell pellets were harvested and total RNA was extracted using TRIzol Reagent (Life Technologies, Grand Island, NY) in accordance with the manufacturer's instructions. cDNA was synthesized with a Reverse Transcription System Kit (Trans Gen Biotech, Beijing, China). Quantitative PCR was carried out with a TransStrat Top Green qPCR SuperMix kit (Trans Gen Biotech) on a real‐time PCR machine (Roche, Pleasanton, CA). The specific mouse and human primers used in this study are summarized in Table S2.

### 2.10 Proliferation assay

The cell growth rate was measured by staining of crystal violet (Beyotime, Shanghai, China). Briefly, cells were seeded at 2 × 10^4^ cells per well in a 6‐well plate. After 2 days, the cells were fixed in methanol and stained with 0.5% crystal violet in 25% methanol for 10 min. The cell plates were dried and then washed in 1% SDS. Cell density was quantified by measuring absorbance of the wash solution at 570 nm using a microplate reader.

### 2.11 Migration assay

A fibroblast migration assay was performed in a Corning BioCoat™ matrigel invasion chamber containing an 8‐μm‐pore size PET membrane coated with a thin layer of extracellular matrix proteins. After coculture with tubular epithelial cells for 48 h, the same number of fibroblasts was seeded in the upper well and cultured for 15 h at 37°C with 5% CO_2_. Non‐invading cells were removed from the upper surface of the chamber and the membrane was stained with crystal violet. Images of the membranes were obtained and cell numbers were measured.

### 2.12 Contractility assay

A fibroblast contraction assay was performed as described previously. A cell suspension was prepared with serum‐free medium mixed with 3 mg/ml type I collagen from rat tail tendon (BD Biosciences, San Jose, CA) at a ratio of 2:1. Then, the cell suspension at a density of 2 × 10^5^ cells/ml was seeded onto a 24‐well plate (300 μl/well). Gels were allowed to polymerize at 37°C for 1 h before adding 1 ml of medium and detaching the edge of gels from the walls of the well. After 48 h, images of the gels were obtained and the gel area was measured using ImageJ software.

### 2.13 Measurement of mitochondrial ROS

Mitochondrial ROS production was detected by MitoSOX (Thermo Fisher Scientific). Briefly, *Lgmn^WT^ and Lgmn* ^KO^ PTECs were seeded at 1500 cells per well in a 96‐well plate. Cells were treated with 250 nM mitoquinone mesylate (Selleckchem, Houston, TX) for 24 h or exosomes for 48 h at 37°C with 5% CO_2_ and then incubated with 5 μM MitoSOX for 15 min at 37°C with 5% CO_2_. The plates were washed with PBS three times and the fluorescence released by MitoSOx was measured at 510 nm (excitation) and 580 nm (emission) using a Clariostar microplate reader.

### 2.14 Measurement of lysosomal pH

Lysosomal pH was measured using Lysosensor Yellow/Blue DND‐100 (Invitrogen). Cells were incubated in medium containing 1 μM Lysosensor for 1 min at 37°C with 5% CO_2_. Fluorescence images were obtained at a wavelength range of 510–641 nm (yellow) and 404–456 nm (blue) under a confocal microscope (Olympus). The yellow and blue fluorescence intensities were measured using ImageJ. The blue/yellow ratio was calculated by a division process in each section. The average blue/yellow ratio of a given sample was calculated in five sections. The intensity was measured in at least three independent experiments.

### 2.15 Electron microscopy

Fresh mouse kidney tissues were cut into approximately 1 × 1 × 3 mm^3^ cube‐like pieces and fixed with 4% precooled glutaraldehyde for at least 2 h and 1% osmium tetroxide for 1–2 h at 4°C and then subjected to standard electron microscopic techniques as described previously. Ultrathin sections (60 nm) were observed under an electron microscopy (model HT7700, Hitachi).

### 2.16 Enrichment of nuclear fractions

Nuclear and cytosolic fractions from shCtrl/shLGMN HK‐2 cells were obtained using the NE‐PER extraction reagents (Thermo Fisher Scientific). Briefly, cells were homogenized in cytosolic extraction reagent (CER I) using a Dounce homogenizer. Homogenates were then vortexed and let to stand on ice for 10 min. Following addition of CER II solution, samples were vortexed and centrifuged for 10 min at 16,000 *g*. The supernatant cytosolic fractions were then collected. The pellets, containing nuclei and cellular debris, were washed in cold PBS and suspended in nuclear extraction buffer (NER). Nuclear fractions were then sonicated three times for ~3 to 5 s at 30% power, and incubated on ice for 40 min. After centrifuged for 10 min at 16,000 *g*, the resulting supernatant nuclear fractions were collected and stored at −80°C for further analysis.

### 2.17 Assessment of autophagic flux

To assess the autophagic flux in young and elderly Lgmn KO and wild‐type mice. Kidneys from 3‐ and 18 months old CAG‐RFP‐EGFP‐LC3 labeled *Lgmn* ^WT^/*Lgmn* ^KO^ mice were collected. Kidney tissues were prepared for frozen sections (5 μm thick). Slides were washed with PBS, then mounted using the mounting medium with DAPI. The fluorescence images were collected using confocal microscope (FV1000, Olympus). GFP and RFP‐MAP1LC3B dots per proximal tubule were counted in at least 10 fields (×400). The intensity of the positive staining area was measured using ImageJ software.

### 2.18 Extraction of soluble and insoluble protein fractions

Extraction of soluble and insoluble proteins was performed as previously described (Festa et al., 2018). Briefly, kidney tissues were lysed in buffer (50 mM pH 7.5 Tris‐HCl, 150 mM NaCl, 0.1% SDS, 1% Triton X‐100, and 1% sodium deoxycholate) supplemented with protease and phosphatase inhibitors, and centrifuged at 13,300 *g* for 20 min at 4°C, to collect the soluble fraction (supernatant). The pellet was suspended in a buffer containing 4% SDS and 20 mM pH 7.5 HEPES, protease and phosphatase inhibitors, then centrifuged at 15,000 *g* for 10 min at room temperature, to collect the insoluble fraction (supernatant). Samples were then prepared for Western blotting analysis.

### 2.19 Mitochondria isolation

To isolate mitochondrial fractions, freshly prepared renal tubules were homogenized using a Dounce homogenizer. Mitochondria were then isolated by differential centrifugation using ice‐cold mitochondria isolation buffer (70 mM sucrose, 200 mM D‐mannitol, 5 mM 3‐N‐morpholino propane sulfonic acid, 2 mM taurine, 1.6 mM carnitine hydrochloride, 1 mM EDTA, 0.025% bovine serum albumin). Mitochondrial samples were collected for subsequent Western blotting procedures.

### 2.20 Exosome isolation and cell treatment assay

Exosomes were purified from conditioned media of control and PTECs transduced with a lentiviral vector that overexpressed legumain (Lgmn OE) or blank control (MCS) separately. Cells were cultured in DMEM/F12 supplemented with 10% exosome‐depleted FBS and 1% P/S. At 90% confluence, the medium was changed to DMEM/F12 and then collected after 48 h. Exosomes were purified by sequential centrifugation at 300 *g* for 10 min, 2,000 *g* for 10 min, and 10,000 *g* for 30 min to remove cell debris. The medium was then ultracentrifuged at 100,000 *g* for 2 h (model X90, Beckman Coulter). Exosome pellets were resuspended in ice‐cold PBS and used immediately or stored at −80°C. All steps were performed at 4°C.

For the cell treatment assay, elderly (18‐month‐old) PTECs were seeded in a 6‐well plate. Cells were cultured in DMEM/F12 complete medium containing legumain overexpressing (Lgmn OE) or control (MCS) exosomes (200 μg) for 48 h at 37°C with 5% CO_2,_ and then, the supernatant was collected for further analysis.

### 2.21 Statistical analysis

All results are presented as the mean ± SEM and analyzed using GraphPad Prism, Version 7.0 (GraphPad Software Inc., La Jolla, CA). Comparisons of two groups were performed using the unpaired Student's *t*‐test. For multiple group comparisons, ANOVA followed by Bonferroni's post hoc correction was used. The correlation between legumain expression in kidney and the level of renal interstitial fibrosis was analyzed by Pearson correlation analysis. *p* < 0.05 was considered statistically significant.

## 3 RESULTS

### 3.1 Aging‐related loss of legumain correlates positively with renal fibrosis

**FIGURE 1:**
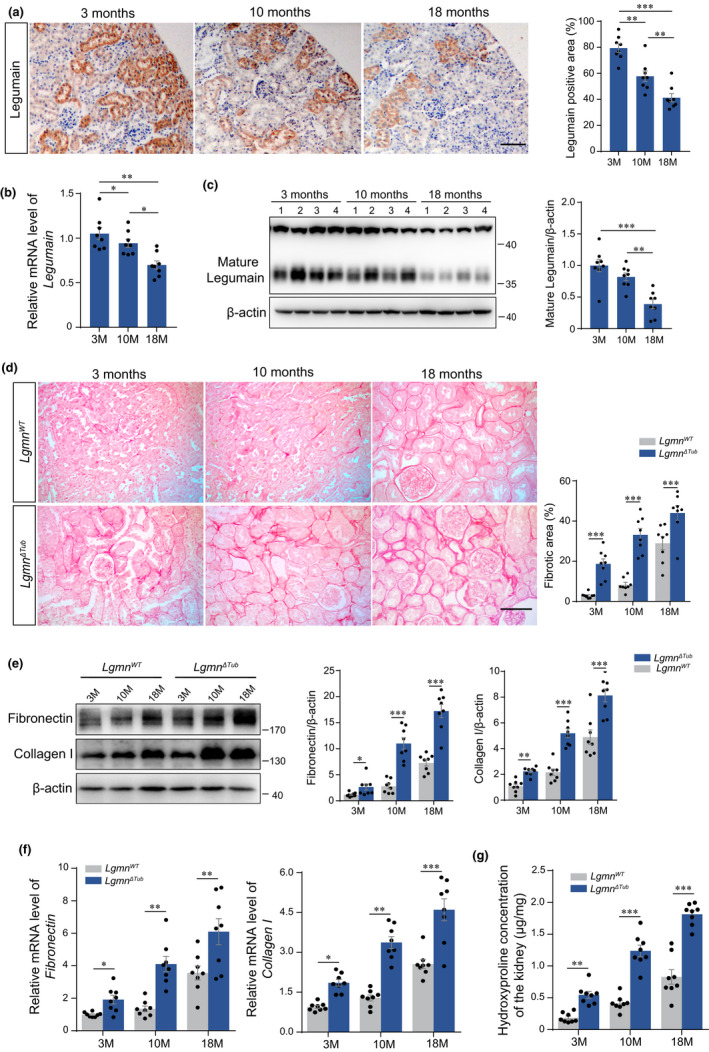
Aging‐related loss of tubular legumain correlates with aggravated renal fibrosis. Kidney samples were collected from C57/BL6J mice at 3, 10, and 18 months of age (*n* = 8). (a) Representative images and quantitative analysis of immunostaining of legumain in kidney sections. Scale bar, 50 μm. (b) Relative legumain mRNA level in whole kidney lysates measured by real‐time PCR. (c) Representative images and quantitative analysis of legumain in whole kidney lysates assessed by Western blotting. Kidney samples were collected from wild‐type and *Lgmn* ^ΔTub^ mice at 3, 10, and 18 months of age (*n* = 8). (d) Representative images and quantitative analysis of Sirius red staining in kidney sections. Scale bar, 50 μm. (e) Representative images and quantitative analysis of fibronectin and collagen I detected by Western blotting. (f) mRNA expression of fibronectin and collagen I detected by real‐time PCR. (g) Collagen volume detected by a hydroxyproline assay in whole kidney lysates. Data are presented as mean ± SEM. ***p < 0.001, **p < 0.01, *p < 0.05

To evaluate the change of legumain in renal tubules during aging, we first measured the expression of legumain in 3‐, 10‐, and 18‐month‐old mice. As shown in Figure 1a, renal tubules were the major sites of legumain expression, and tubular expression of legumain was decreased with aging. Analysis of semi‐quantitative PCR and Western blot confirmed that expression of legumain was decreased in the elderly kidney (Figure 1b,c), which implied its role in the aging‐related tubular lesion. We next compared the extent of renal interstitial fibrosis in the kidneys of wild‐type (WT) and proximal tubular‐specific legumain‐knockout (*Lgmn* ^ΔTub^) mice. Sirius red staining demonstrated gradually increased accumulation of the extracellular matrix in WT and *Lgmn* ^ΔTub^ mice with aging. Detectable interstitial fibrosis appeared at 3 months after birth in *Lgmn* ^ΔTub^ mice and severer renal fibrosis could be observed at the 10‐ and 18‐month *Lgmn* ^ΔTub^ mice (Figure 1d). Expression of fibronectin and collagen I at both mRNA and protein level (Figure 1e,f), and collagen volume assessed via hydroxyproline assay (Figure 1g) confirmed the results of Sirius red staining. In order to address whether the levels of other lysosomal proteases also declined with age, we examined the protein expression of cathepsins B and cathepsins D in kidney tissue by Western blot. Our results showed that compared with young mice, expression of cathepsins B in the renal homogenate is increased in the elderly mice, and no significant changes were observed for the level of cathepsins D (Figure S1A). We then compared the level of cathepsins B, cathepsins D, and LGMN in different tissues through analyzing the dataset obtained from National Center for Biotechnology Information (NCBI). It is revealed the distinct tissue‐specific expressional pattern for cathepsin D, cathepsin B, and LGMN (Figure S1B). Together, these data suggest the unique role of LGMN in the aging‐related fibrotic lesions.

In human samples, we performed immunohistochemical staining and protein analysis of legumain in para‐cancerous normal kidney sections obtained from patients of different ages with renal carcinoma. As shown in Figure S2A,B, expression of legumain was significantly downregulated in elderly human kidneys. Sirius red staining demonstrated more serious renal interstitial fibrosis in elderly kidneys (Figure S2C). Moreover, negative correlation was exhibited between tubular expression of legumain and the extent of renal interstitial fibrosis in human kidney samples (Figure S2D).

### 3.2 Deficiency of legumain induces premature senescence and drives renal fibrosis

**FIGURE 2:**
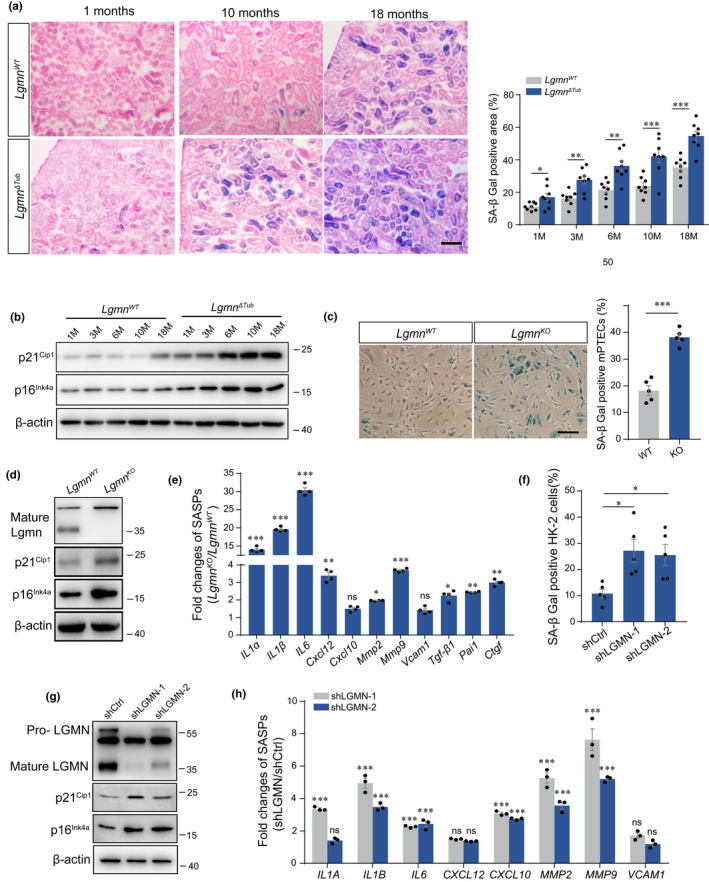
Deficiency of legumain accelerates senescence of renal tubular cells. Kidney samples were collected from wild‐type and *Lgmn* ^ΔTub^ mice at 1, 3, 6, 10, and 18 months of age (*n* = 8). (a) Representative images and quantitative analysis of SA‐β‐gal activity in kidney sections. Scale bar, 50 μm. (b) Western blot analysis of p21^Cip1^ and p16^Ink4a^ in whole kidney lysates. Primary mouse renal tubular epithelial cells (PTECs) were isolated from wild‐type and *Lgmn* ^ΔTub^ mice of 3‐month‐old. (c) Representative images and quantitative analysis of SA‐β‐gal activity assay. Scale bar, 50 μm. (d) Western blot analysis of legumain, p21^Cip1^, and p16^Ink4a^ in PTECs. (e) Fold change of mRNA expression of SASP factors (Il‐1α, Il‐1β, Il‐6, Cxcl12, Cxcl10, Mmp2, Mmp9, Vcam1, Tgf‐β1, Pai‐1, and Ctgf). Stable Scrambled control shRNA (shCtrl)‐ and legumain shRNA (shLGMN)‐transfected HK‐2 cells were established. (f) Analysis of SA‐β‐gal activity. (g) Western blot analysis of legumain, p21^CIP1^, and p16^INK4A^. (h) Fold change of mRNA expression of SASP factors (IL‐1α, IL‐1β, IL‐6, CXCL12, CXCL10, MMP2, MMP9, VCAM1, TGF‐β1, PAI‐1, and CTGF). Data are presented as mean ± SEM. ***p < 0.001, **p < 0.01, *p < 0.05

Accumulation of senescent cells has been observed in multiple elderly tissues and cellular senescence is thought to be a major driver of age‐associated diseases. We then compared the senescent indexes, which included SA‐β‐gal activity and major senescence‐related signaling molecules p21^Cip1^and p16^Ink4a^ in kidneys of WT and *Lgmn* ^ΔTub^ mice. Our results showed that significantly increased activity of SA‐β‐gal was observed in 1‐, 3‐, 6‐, 10‐, and 18‐month kidneys of *Lgmn* ^ΔTub^ mice compared with that of the control (Figure 2a). Consistently, increase of senescence master regulators p21^Cip1^ and p16 ^Ink4a^ had appeared earlier and severer in *Lgmn* ^ΔTub^ mice, which indicated acceleration of premature senescence (Figure 2b). We also isolated the primary tubular epithelial cells (PTECs) of 3‐month‐old mice and measured the level of cellular senescence. Similar to the results obtained from tissue lysates, primary *Lgmn* ^ΔTub^ tubular cells exhibited increased in activity of SA‐β‐gal, expression of p21^Cip1^ and p16^Ink4a^, and the senescence‐associated secretory phenotype (SASP) compared with the control (Figure 2c–e). We then constructed stable legumain‐knockdown and mock control HK‐2 cells to explore whether downregulation of legumain induces senescence in tubular cells. As shown in Figure 2f–h, silencing legumain significantly increased the activity of SA‐β‐gal, expression of p21^CIP1^, p16^INK4A^, and SASPs.

**FIGURE 3:**
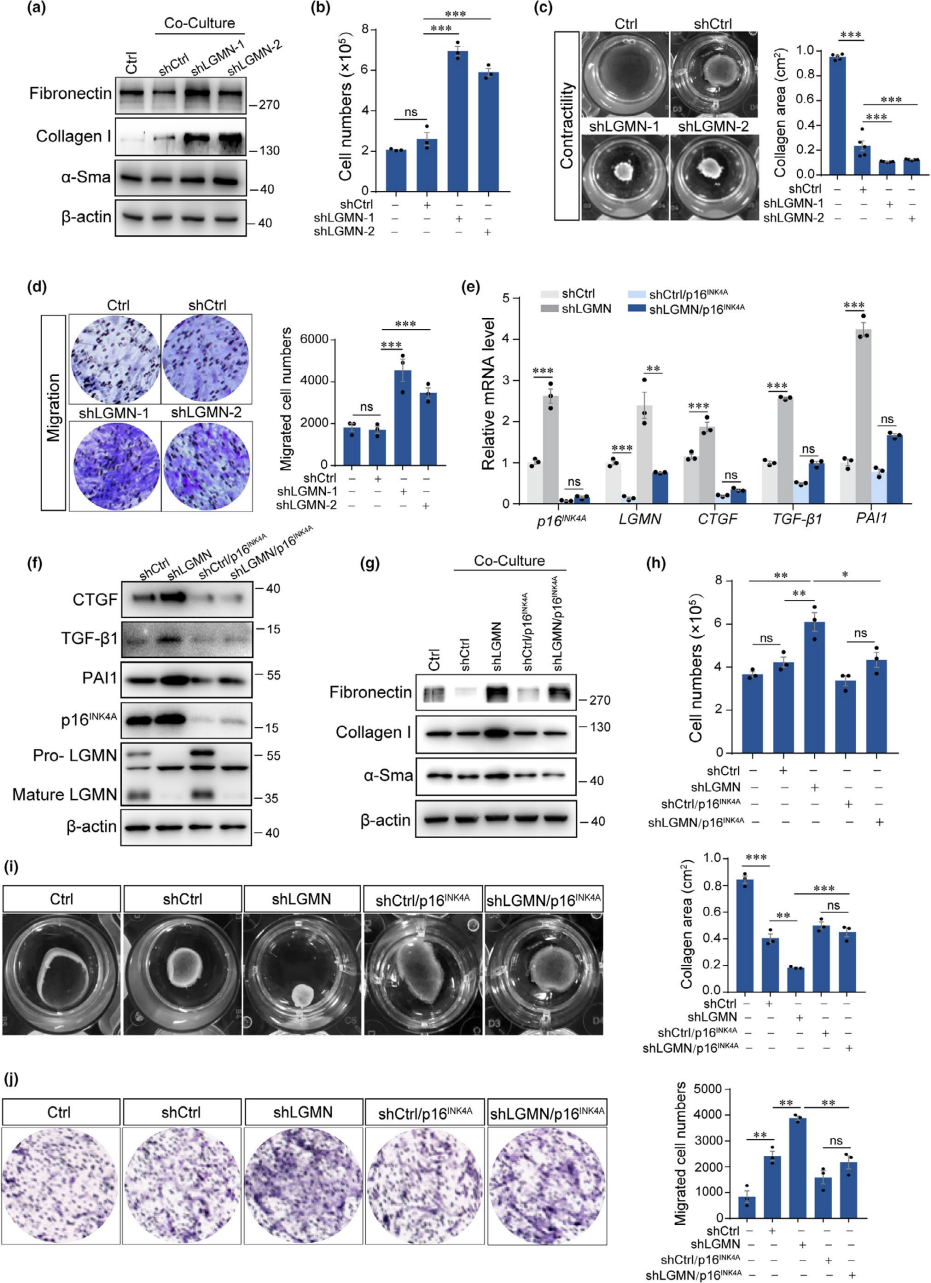
Senescence cell‐derived cytokines promote fibrosis in legumain‐deficient kidneys. Stable shCtrl and shLGMN HK‐2 cells were established as described in Figure 2f and then cocultured with fibroblasts in a non‐contact system for 48 h. (a) Western blot analysis of fibronectin, collagen I, and α‐SMA expression in fibroblasts. (b) Cell counting of fibroblasts detected by crystal violet staining. (c) Representative images and quantitative analysis of fibroblast contractility detected by three‐dimensional collagen gel assays. (d) Representative images and quantitative analysis of fibroblast migration detected by transwell assays. p16^INK4A^ was downregulated in shCtrl and shLGMN HK‐2 cells and then the cells were cocultured with fibroblasts for 48 h, respectively. (e, f) Western blotting and real‐time PCR analysis of p16^INK4A^, LGMN, CTGF, PAI‐1, and TGF‐β1expression. (g) Western blot analysis of fibronectin, collagen I, and α‐SMA expression. (h) Cell counting of fibroblasts detected by crystal violet staining. (i) Representative images and quantitative analysis of fibroblast contractility detected by three‐dimensional collagen gel assays. (j) Representative images and quantitative analysis of fibroblast migration detected by transwell assays. Data are presented as the mean ± SEM. ***p < 0.001, **p < 0.01, *p < 0.05

To evaluate the effect of downregulation of legumain on renal fibrosis, we used a non‐contact coculture system of HK‐2 cells and BJ fibroblasts. Analysis of fibrotic proteins revealed that coculture of legumain‐downregulated HK‐2 cells induced expression of fibronectin, collagen I, and α‐SMA in fibroblasts (Figure 3a). Cell counting showed that coculture with stable legumain‐silenced HK‐2 cells promoted the proliferation of fibroblasts (Figure 3b). We also performed contractility and migration assay via three‐dimensional collagen gel and transwell assay separately. As shown in Figure 3c,d, coculture with legumain‐downregulated HK‐2 cells increased the collagen contractility and migration capacity of BJ cells compared with the mock control. Next, knockdown of *p16^INK4A^* was used to confirm the role of senescence in the profibrotic effect of legumain downregulation. Pro‐fibrotic cytokines CTGF, TGF‐β1, and PAI‐1, which are also typical SASP factors, were assessed. As shown in Figure 3e,f, knockdown of *p16^INK4A^* suppressed legumain downregulation‐induced expression of CTGF, TGF‐β1, and PAI‐1 at both protein and mRNA level. Consistently, knockdown of *p16^INK4A^* rescued activation of fibroblasts after coculture of legumain‐downregulated HK‐2 cells as indicated by expression of fibrotic proteins, cell counting as well as contractility and migration assay (Figure 3g–j).

### 3.3 Legumain participates in the maintenance of lysosomal homeostasis

**FIGURE 4:**
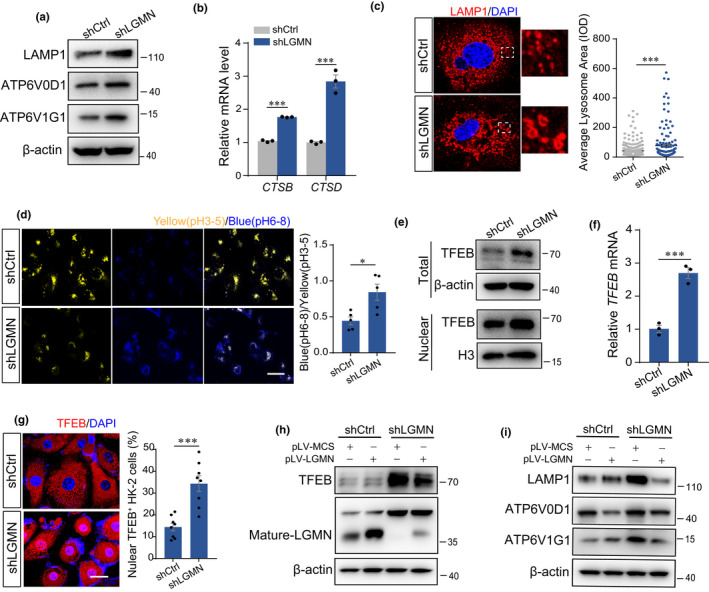
Legumain deficiency activates TFEB and disrupts lysosomal homeostasis. Stable shCtrl and shLGMN HK‐2 cells were established as described in Figure 2f. (a) Western blot analysis of lysosomal markers LAMP1, ATP6V0D1, and ATP6V1G1 in shCtrl and shLGMN HK‐2 cells. (b) Real‐time PCR analysis of CTSD and CTSB in cultured HK‐2 cells. (c) Immunofluorescence staining and quantitative analysis of LAMP1 in cultured cells. Scale bar, 10 μm. (d) Representative images and quantitative analysis of cells detected by LysoSensor Yellow/Blue. Scale bar, 50 μm. (e) Western blot analysis of TFEB in whole‐cell lysates and the cell nucleus. (f) Real‐time PCR analysis of TFEB in whole‐cell lysates. (g) Immunofluorescence staining and quantitative analysis of TFEB in cultured cells. Scale bar, 20 μm. shCtrl and shLGMN HK‐2 cells were transiently transduced with a virus that overexpressed legumain. (h, i) Western blot analysis of legumain, TFEB, LAMP1, ATP6V0D1, and ATP6V1G1 in cell lysates. Data are presented as the mean ± SEM. ***p < 0.001, **p < 0.01, *p < 0.05

Legumain acts majorly in lysosomes and belongs to a unique enzyme family that cleaves after asparagine with high specificity. Therefore, we investigated the effect of legumain deficiency on the status of lysosomes. Lysosomal proton‐transporting V‐type ATPase (V‐ATPase) is responsible for acidifying lysosomes, which is regulated through diverse subunits of integral membrane channel complex. As shown in Figure 4a, Lysosomal protein LAMP1 and v‐ATPase, ATP6V0D1, and ATP6V1G1 were increased in legumain‐knockdown HK‐2 cells compared with the control.

Expression of major lysosome endopeptidases CTSD and CTSB was induced by downregulation of legumain (Figure 4b), which suggested increased biogenesis of lysosomes. Staining of lysosome tracker LAMP1 (Figure 4c) and lysosomal pH value analysis (Figure 4d) showed enlargement of lysosome size and increase lysosomal pH in legumain‐knockdown HK‐2 cells compared with the control, which indicated disturbance of the lysosomal homeostasis. Nuclear transcription factors EB (TFEB) governs transcription of lysosomal biogenesis genes. We therefore accessed the expression and nuclear translocation of TFEB. The results showed that knockdown of legumain promoted the expression and activation of TFEB in tubular cells (Figure 4e–g). Overexpression of legumain abolished the increase of TFEB and expression of its target genes in the legumain‐knockdown HK‐2 cells (Figure 4h,i). Taken together, our data suggested that downregulation of legumain promoted activation of TFEB and disrupted lysosomal homeostasis.

### 3.4 Legumain deficiency in elderly tubular cells stagnates autophagic flux

**FIGURE 5:**
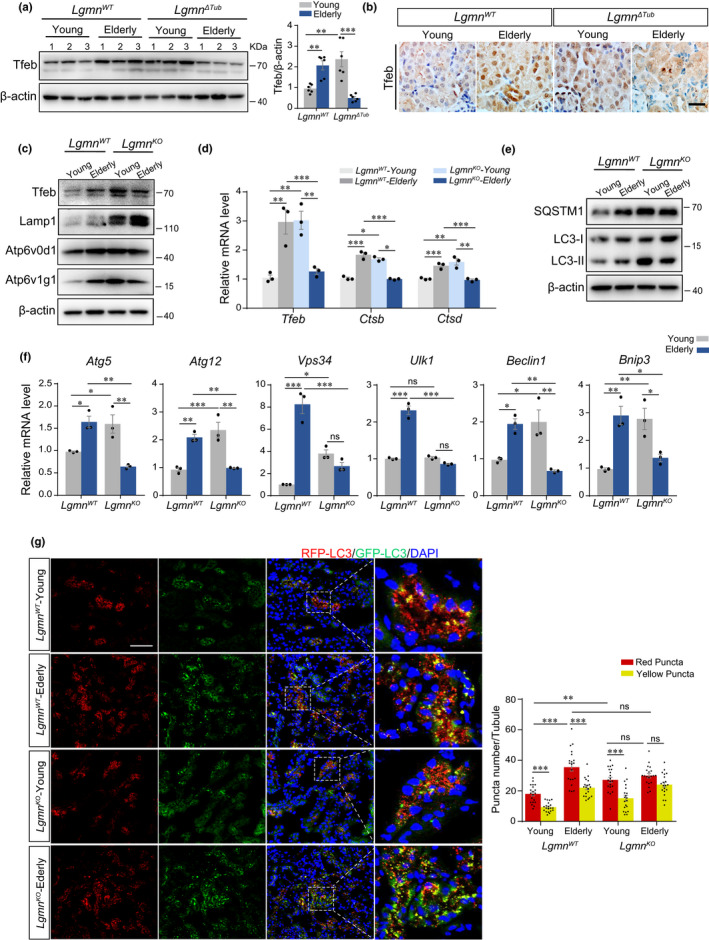
Deficiency of legumain disturbs aging‐induced lysosomal homeostasis. Kidney samples were collected from wild‐type and *Lgmn* ^ΔTub^ mice of young (3‐month‐old) and elderly (18‐month‐old) (*n* = 6). (a) Western blotting and quantitative analysis of Tfeb in the kidney. (b) Immunohistochemistry staining of Tfeb in the kidney slides. Scale bar, 20 μm. PTECs were isolated from wild‐type and legumain‐knockout mice elderly 3 and 18 months. (c) Western blot analysis of Tfeb, Lamp1, Atp6v0d1, and Atp6v0d1 in the cell lysates. (d) Real‐time PCR analysis of Tfeb, Cathepsin D, and Cathepsin B in the cultured cells. (e) Western blot analysis of p62/Sqstm1 and LC3‐I/II in the cell lysates. (f) Real‐time PCR analysis of mRNA expression of Atg5, Atg12, Vps34, Beclin1, Bnip3, and Ulk1 in the cultured cells. Conventional legumain‐knockout (LgmnKO) mice with RFP‐EGFP‐LC3 fluorescent tag were established. (g) Kidney samples were collected from *Lgmn* ^KO^ and *Lgmn* ^WT^ RFP‐EGFP‐LC3 transgenic mice of young (3‐month‐old) and elderly (18‐month‐old) (*n* = 3). Representative images of fluorescent microscopy of renal frozen slides. Scale bar, 50 μm. Data are presented as the mean ± SEM. ***p < 0.001, **p < 0.01, *p < 0.05

Given the influence of legumain deficiency on the status of lysosomes, we then explored whether change of lysosomal status plays a role in the profibrotic effect of legumain deficiency. We measured the expression level of TFEB in the young and elderly kidneys of WT and *Lgmn* ^ΔTub^ mice separately. Western blotting and immunohistochemistry assay showed that TFEB expression was increased in *Lgmn* ^ΔTub^ mice at young age. In WT mice, TFEB was increased in elderly mice compared with the young mice. Unexpectedly, activation of TFEB was significantly downregulated in elderly *Lgmn* ^ΔTub^ mice compared with all other groups (Figure 5a,b). A similar change in the expression of TFEB was found in renal tubular cells isolated from the mouse models. Indexes of lysosomal status, Lamp1, Atp6v0d1, Atp6v1g1, CtsB, and CtsD were increased in legumain‐knockout and elderly wild‐type PTECs, while there was no further increase in elderly legumain‐knockout tubular cells (Figure 5c,d).

In addition to degradation on substances, lysosomes participate in multiple aspects of cell and tissue physiologies including autophagy, a major intracellular degradation system related closely to aging and age‐related lesions. We therefore evaluated the effect of legumain‐knockout on the initiation and processing of autophagy. Our data showed that the ratio of LC3‐II/LC3‐I was increased in young legumain‐knockout or elderly WT tubular cells, which indicated activation of autophagy.

However, p62/sqstm1 was also increased, which suggested stagnant autophagic flux. In elderly legumain‐knockout tubular cells, increased p62/sqstm1 had remained, although LC3‐II/LC3‐I was downregulated compared with WT and young legumain‐knockout controls (Figure 5e). Expression of several critical autophagy‐related genes, *Atg5*, *Atg12*, *Vps34*, *Beclin1*, *Bnip3*, and *Ulk1* also showed similar pattern, namely elderly or legumain‐knockout cells had increased expression of these autophagy‐related genes, but it did not further increase in elderly legumain‐knockout tubules (Figure 5f). To visualize the change of autophagic flux, we manipulated the *Lgmn^WT^* /*Lgmn^KO^* RFPGFP‐LC3 transgenic mice constructed in our laboratory. Fluorescence microscopy assay revealed that maximal stagnant of autophagic flux (yellow/red) occurs in the tubule of elderly legumain‐knockout group, suggesting the incompetent response to lysosome stress in the condition (Figure 5g).

### 3.5 Loss of legumain impairs mitophagy and increases aging‐related mtROS accumulation

**FIGURE 6:**
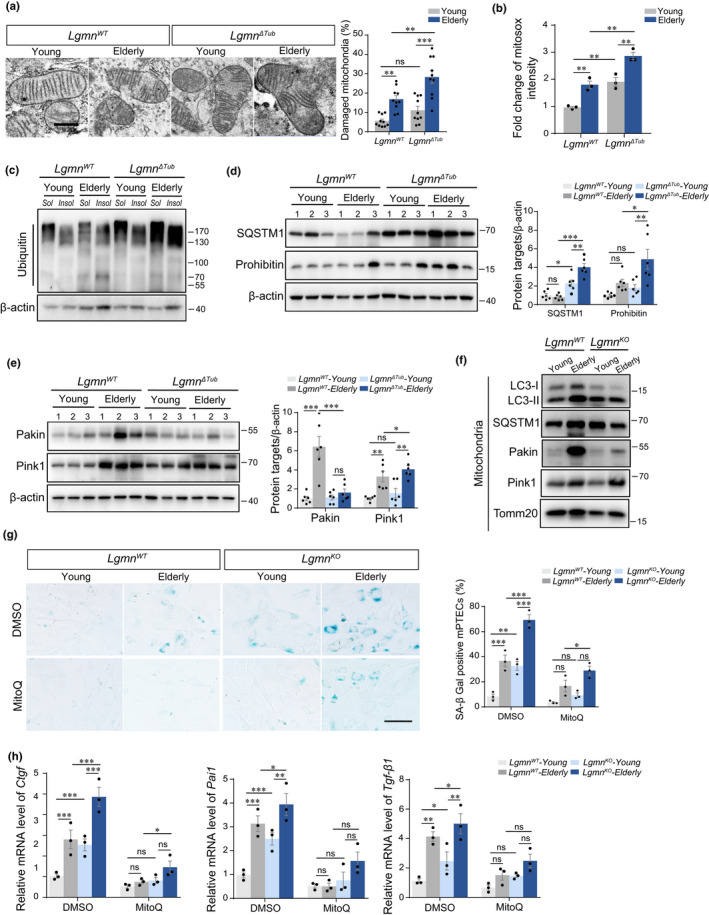
Deficiency of legumain promotes mtROS accumulation and profibrotic effect of senescence in the elderly tubule. Kidney samples were collected from wild‐type and *Lgmn* ^ΔTub^ mice of young (3‐month‐old) and elderly (18‐month‐old) (*n* = 3). (a) Representative images and quantitative analysis of mitochondria morphology detected by transmission electron microscopy. Scale bar, 1 μm. PTECs were isolated from wild‐type and legumain‐knockout mice aged 3 or 18 months. (b) Quantitative analysis of mitochondrial ROS detected by the MitoSOX probe. (c) Soluble and insoluble protein fractions of the kidney were extracted from young and elderly wild‐type/*Lgmn* ^ΔTub^ mice. Western blot analysis of ubiquitin in the fractions. (d) Proteins were extracted from the kidney homogenates of young and elderly wild‐type/*Lgmn* ^ΔTub^ mice (*n* = 6). Western blot and quantitative analysis of SQSTM1 and prohibitin in the kidneys. (e) Western blot and quantitative analysis of Parkin and Pink1 in the kidneys. (f) Mitochondria was isolated from freshly prepared renal tubules collected from wild‐type and *Lgmn* ^ΔTub^ mice at 3 and 18 months of age. Western blotting of LC3‐I/II, SQSTM1, Parkin, and Pink1. Tomm20 was used as internal control for the loading volume of mitochondria. (g) Representative images and quantitative analysis of SA‐β‐gal activity in PTECs after treatment with MitoQ. Scale bar, 50 μm. (h) Real‐time PCR analysis of Ctgf, Pai‐1, and Tgf‐β1 in PTECs after treatment with MitoQ. Data are presented as the mean ± SEM. ***p < 0.001, **p < 0.01, *p < 0.05

Mitochondria are a major intracellular source of ROS, and mitochondrial damage increases mtROS production. Damaged mitochondria are effectively removed via mitophagy, thereby maintaining mitochondrial function. To identify the role of legumain loss in mitophagy and accumulation of mtROS during aging, we examined the effect of legumain deficiency on mitophagy. TEM revealed more mitochondrial damage in legumain‐knockout kidney (Figure 6a). Dysfunctional mitochondria are the major source of excessive ROS production that causes the acceleration of senescence. We therefore hypothesized that the ROS generated due to damaged mitophagy mediated the acceleration of senescence induced by legumain deficiency. Levels of mtROS were measured by using ROS fluorescent probe mitoSOX. Both downregulation of legumain and aging increased mtROS and these effects reached peak in elderly legumain‐knockout tubular cells (Figure 6b). To evaluate the pattern of autophagy substrates accumulated in the elderly and young *Lgmn* ^ΔTub^ tissue, we accessed the ubiquitinated soluble/insoluble proteins in the kidney tissue homogenates. Our results showed significant increased insoluble ubiquitin‐positive fractions in the elderly *Lgmn* ^ΔTub^ kidneys compared with young *Lgmn* ^ΔTub^ ones (Figure 6c). Level of SQSTM1, as well as prohibitin (an inner mitochondrial membrane protein), was detected via Western blotting. Our results suggest aggregate‐forming SQSTM1 and ubiquitin‐positive inclusions. Moreover, increased level of prohibitin suggests that disruption of autophagy led to the accumulation of dysfunctional mitochondria in the elderly *Lgmn* KO kidneys compared with young *Lgmn* KO ones (Figure 6d). In order to strengthen the link between dysfunctional autophagy and mitochondrial defects, we measured the level of mitophagy markers PINK1/Parkin in the kidney homogenates of wild‐type and *Lgmn* ^ΔTub^ mice at 3 and 18 months of age. We also isolated mitochondria from freshly prepared renal tubules collected from wild‐type and *Lgmn* ^ΔTub^ mice at 3 and 18 months of age to measure the mitophagy. Western blotting was performed using the antibody against mitophagy markers, PINK1and Parkin. Consistent with the data on autophagy, our data showed that mitophagy pathway PINK1 and Parkin are increased in the elderly WT compared with young WT control, while significantly suppressed in the either young or elderly KO ones (Figure 6e,f). Consistently, the highest level of SA‐β‐gal activity was seen in elderly legumain‐knockout tubular cells, which was rescued by application of mtROS scavenger MitoQ (Figure 6g). Moreover, legumain‐knockout‐induced expression of profibrotic factors *Pai*‐*1*, *Ctgf*, and *Tgf*‐*β1* in young legumain‐knockout, elderly WT, and elderly legumain‐knockout tubular cells was similarly decreased with the clearance of mtROS (Figure 6h).

### 3.6 Exosomal legumain alleviates premature senescence and ameliorates fibrosis

**FIGURE 7:**
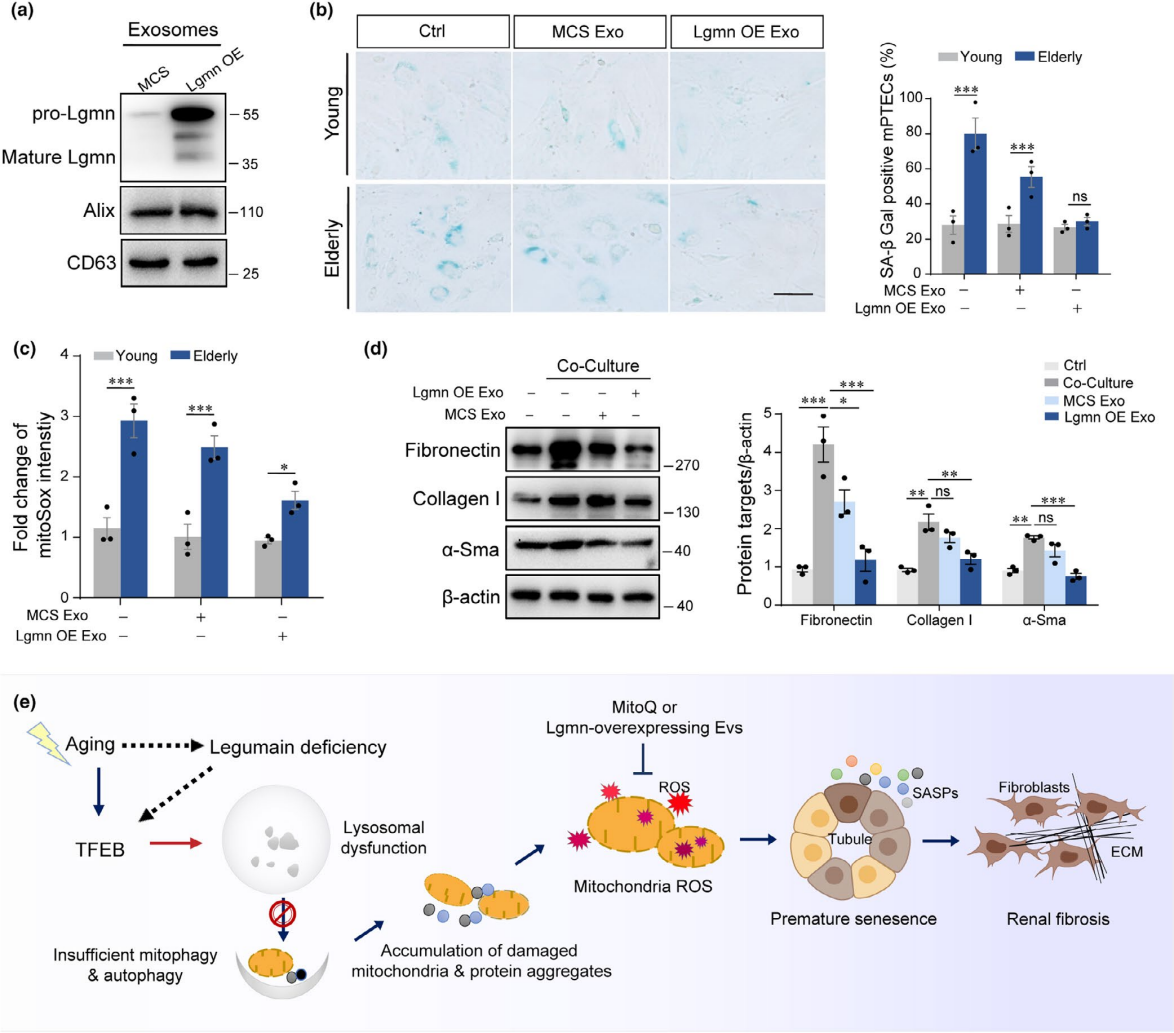
Supplementation of legumain alleviates senescence and inhibits the profibrotic effect of elderly PTECs. Legumain‐overexpressing EVs were prepared from Lgmn‐overexpressing PTECs by ultracentrifugation. (a) Western blot analysis of legumain in legumain‐overexpressing (Lgmn OE) and control (MCS) EVs. Expression of Alix and CD63 was used as internal control. PTECs were isolated from young (3‐month‐old) and elderly (18‐month‐old) wild‐type mice and then treated with legumain‐overexpressing or control EVs for 48 h. (b) Representative images and quantitative analysis of SA‐β‐gal activity in cultured cells. Scale bar, 50 μm. (c) Quantitative analysis of mitochondrial ROS detected by the MitoSOX probe. (d) Elderly PTECs were treated with Lgmn OE or MCS EVs for 48 h and then conditioned medium was collected after culture for another 24 h. MEFs were treated with the CMs described above for 48 h, and then, cell lysates were collected for analysis. Western blot and quantitative analysis of fibronectin, collagen I, and α‐SMA expression. Data are presented as the 783 mean ± SEM. ***p < 0.001, **p < 0.01, *p < 0.05. (e) Schematic illustration of the hypothesized mechanism of aging‐related renal fibrosis

Next, we made the preliminary attempt at the treatment of legumain supplementation against aging‐related renal fibrosis. Exosomes derived from legumain‐overexpressing primary mouse renal tubular cells were used as the resource of legumain supplementation. First, we constructed legumain‐ or MCS‐overexpressing tubular cells, respectively. Legumain could be detected in the exosomes collected from the legumain‐overexpressing tubular cells (Figure 7a). Application of legumain‐overexpressing exosomes to young or elderly legumain‐knockout tubular cells suppressed the level of cellular senescence as shown by SA‐β‐Gal activity (Figure 7b). Quantitative analysis of mtROS by MitoSOX probe showed similar results. Legumain‐overexpressing exosomes decreased mtROS in young and elderly legumain‐knockout tubular cells (Figure 7c). We collected conditioned medium (CM) from elderly tubular cells treated with legumain‐overexpressing exosomes or control exosomes, and then cultured myofibroblasts with the CMs, separately. Western blot analysis of fibrosis‐associated proteins fibronectin, collagen I, and α‐SMA showed that the most dramatic relief of fibrosis had occurred in the group treated with CMs from elderly legumain‐knockout tubular cells with legumain‐overexpressing exosomes (Figure 7d). Altogether, our data showed that lysosomal stress caused by either loss of legumain or aging boosts the function of autophagy‐lysosome system, while the overlapping the challenges of aging and legumain deficiency leads to the perturbation of this system and then an accumulation of damaged mitochondria and the activation of downstream pathway (Figure 7e).

## 4 DISCUSSION

Progressive CKD and aging kidneys share many similarities in both manifestation and underlying molecular mediators, which suggests that CKD is a clinical model of premature kidney aging (Zhou et al., 2008). Tubular interstitial fibrosis, the common histological feature of end‐stage renal diseases, appears to be prominent among the age‐related kidney structural changes (Docherty et al., 2019). SASP and the CKD‐associated secretory phenotype, the loss of renoprotective factors Klotho and bone morphogenetic proteins, vascular rarefaction, and oxidative stress are similar pathologic events involved in both aging and CKD (O'Sullivan et al., 2017). Recent studies have revealed a cell senescence intersection of the two entities. Diverse kidney stressors induce cell senescence, resulting in the tendency toward chronicity of renal disease due to the release of proinflammatory and profibrotic components of SASP. Moreover, insidious accumulation of senescent cells in the aging kidney makes it susceptible to ischemic and nephrotoxic insults that stimulate accumulation of stress‐induced premature senescence with acute‐on‐chronic release of SASP factors, which leads to renal inflammation, fibrogenesis, and functional deterioration (Clements et al., 2013). Elimination of senescent cells has been demonstrated to improve longevity and organ functions. Additionally, rejuvenation of senescent cells by pharmacological methods is capable of reversing organ dysfunction (Chondrogianni et al., 2010; Lu et al., 2010). On the basis of the release of SASP factors with propagating cell dysfunction, attempts to use stem cell therapies and *ex vivo* engineered organs have been made (Bernet et al., 2014; Pricola et al., 2009). In this study, we identified loss of legumain promotes aging‐related renal fibrosis by accelerating tubular cell senescence and consequent activation of fibroblasts. Supplementation of legumain alleviated premature senescence and ameliorated aging‐related renal fibrosis. Considering the similarities between kidney aging and CKD, application of Legumain to the treatment of CKD is anticipated, although further experiments are obviously warranted.

The status of renal tubules correlates closely with maintenance of lysosome homeostasis because of its heavy load of proteins and largely reliance on autophagy (Shamekhi Amiri, 2019). Lysosomes are located at the terminal process of the autophagic pathway and play a crucial role in autophagic degradation of macromolecules and organelles (Fougeray & Pallet, 2015). A growing body of evidence has indicated autophagy as a protective element for epithelial renal cells under healthy, aging, and disease conditions (Isaka et al., 2011; Jiang et al., 2012; Kimura et al., 2011; Takahashi et al., 2017). It modulates tissue responses during acute kidney injuries and protects against aging‐related renal disorders. Our data demonstrated that loss of legumain induced the expression of lysosomal proton‐transporting V‐type ATPase and disrupted acidification of the lysosomal lumen. Additionally, enlargement of the lysosome size and an increase of p62/sqstm1 in legumain‐knockout tubular cells suggested a stagnancy of autophagic flux. Martinez et al have reported that loss of legumain induces expression of multiple other lysosomal proteases and compensates for lysosomal functions (Martinez‐Fabregas et al., 2018). In this study, we also observed increased CtsD and CtsB upon depletion of legumain in tubular cells. However, we found dampened autophagic flux in the legumain‐knockout tubules, especially in elderly ones. This discrepancy raised the issue of whether maintenance of lysosomal function achieved depends on context, the cellular type, or a distinct functional status.

Compelling evidence indicates that lysosomes adapt to different environmental cues and this “lysosomal adaptation” has been significantly implicated in health, aging, and diseases (Ballabio & Gieselmann, 2009; Rajawat et al., 2009; Settembre et al., 2012). It remains unclear how lysosomal functions vary in different cells, tissues, life stages, and individuals, as well as under different physiological conditions. Recent studies have revealed that TFEB acts as a master regulator of cellular degradative pathways, positively regulates the expression of lysosomal genes, controls the number of lysosomes, and promotes the ability of cells to degrade lysosomal substrates (Sardiello et al., 2009). Moreover, TFEB modulates proteins involved in degradation of known autophagy substrates and overexpression of TFEB induces autophagy, which suggest its critical role in regulating autophagy (Settembre et al., 2011). TFEB is activated during the lysosomal response, which is essential to prevent oxalate nephropathy (Nakamura et al., 2020). Our data demonstrated that legumain deficiency or aging activated TFEB in tubular cells, whereas downregulated instead of further increased activation of TFEB was observed in elderly legumain‐knockout cells, which was consistent with the tendency of lysosomal biogenesis and autophagy. A putative explanation was the failure of the lysosomal adaptive response due to overload of lysosome stress. Aging is the chronic and persistent stimuli of lysosomal stress in terms of a heavy protein load and highly autophagy‐dependent tubular cells. Activation of TFEB during aging promotes lysosomal functions to maintain homeostasis. However, it also weakens the capacity of the lysosomal response when sudden and additional lysosome stress occurs.

Mitochondria are widely involved in cellular senescence as the major generator of ROS that play major roles in senescence by inducing genomic damage, accelerating telomere shortening, and acting as drivers of signaling networks important for maintenance of the senescent phenotype (Chan, 2020). Moreover, mitochondria are required for pro‐oxidant and proinflammatory effects of cellular senescence, which suggest their role as drivers of age‐related diseases (Sun et al., 2016). Maintenance of mitochondrial homeostasis depends on adequate mitophagy, a selective form of autophagy, which selectively removes redundant or damaged mitochondria (Ashrafi & Schwarz, 2013). Timely elimination of abnormal mitochondria in renal tubular cells represents an important quality control mechanism for cell homeostasis and survival during kidney injury and repair (Yamamoto et al., 2016). Although impaired mitophagy and consequent ROS accumulation have been associated with age‐dependent mitochondrial degeneration, treatment of ROS elimination remains controversial because of the dual role for ROS in cell physiology (Bratic & Larsson, 2013). Specifically, high concentrations of ROS are pathological, whereas moderate amounts of ROS are essential to maintain several biological processes including gene expression. In this study, our data demonstrated that significantly increased mtROS had occurred in elderly legumain‐knockout tubular cells with stagnant autophagic flux. A scavenger of mtROS effectively alleviated premature senescence and thus ameliorated fibrosis, which highlighted that mitochondrial ROS may be a decisive target in aging‐related diseases.

Taken together, our data demonstrate that lysosomal proteinase legumain actively participates in the maintenance of lysosome homeostasis during aging of renal tubules. Loss of legumain in elderly tubular cells stagnates autophagic flux, impairs mitophagy, and activates senescence via accumulation of mtROS. Accelerated tubular senescence consequently promotes aging‐related renal interstitial fibrosis. Therefore, this study reveals the potential role of legumain in aging‐related renal fibrosis and sheds light on the treatment of CKD.

## CONFLICT OF INTEREST

The authors declare no competing interests.

## AUTHOR CONTRIBUTIONS

X.T. and D.W. conceived and designed the study. D.W., C.C., L. K., J.G., L.D., D.Z., and X.M. performed the experiments. M.Z., G.L., S.L., and Y.Z. provided key reagents and revised the paper. X.T and D.W. analyzed the data and wrote the manuscript. All the authors approved the final version of the manuscript, discussed the results, and approved the final version of the manuscript.

## Supporting information

Supplementary Material

## Data Availability

The data that support the findings of this study are available from the corresponding author upon reasonable request.
