# Supplementary material for: Loss of legumain induces premature senescence and mediates aging‐related renal fibrosis
Source: Aging Cell. 2022 Feb 23;21(3):e13574. doi: 10.1111/acel.13574 (PMC8920435; doi:10.1111/acel.13574)
Supplement: Supplementary file 1 — Supplementary Material [file ACEL-21-e13574-s001.pdf]

Supplemental Table of Contents

Supplemental Figure 1
Aging-related expression and tissue-specific expressional pattern of CtsB, CtsD and LGMN.

Supplemental Figure 2
Aging-related loss of LGMN were correlated with renal fibrosis in human.

Supplemental Table 1
Personal Information for Human Samples

Supplemental Table 2
Primers for Quantitative PCR

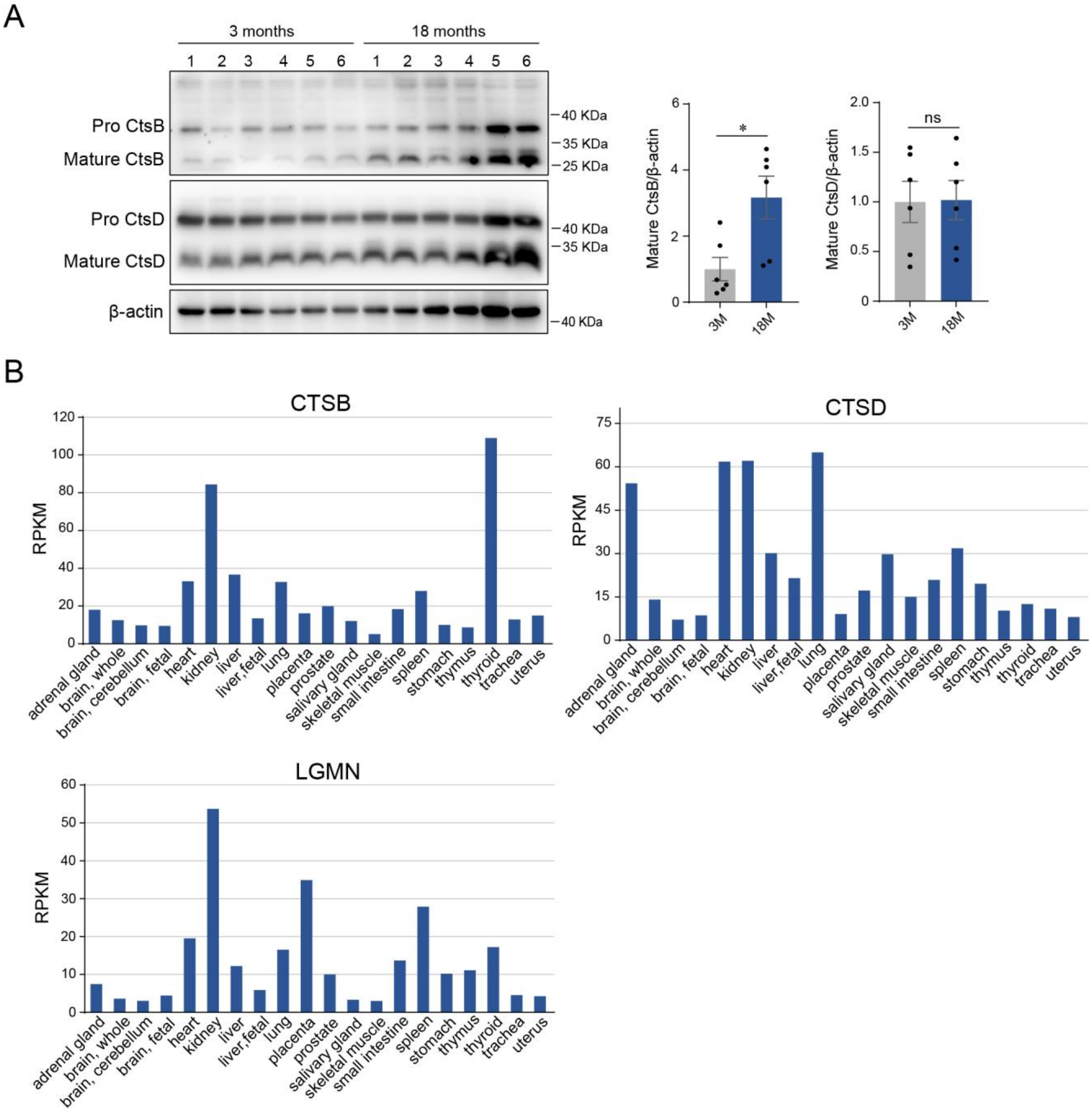

Supplemental Figure 1. Aging-related expression and tissue-specific expressional pattern of CtsB, CtsD and LGMN. Kidney samples were collected from C57/BL6J mice at 3 and 18 months of age (n = 6). (A) Representative images and quantitative analysis of cathepsins B and cathepsins D in whole kidney lysates assessed by western blotting. (B) Tissue specific expression of CtsD, CtsB and LGMN via analysis on dataset from NCBI. Data are presented as mean ± S.E.M. $*p < 0.05$.

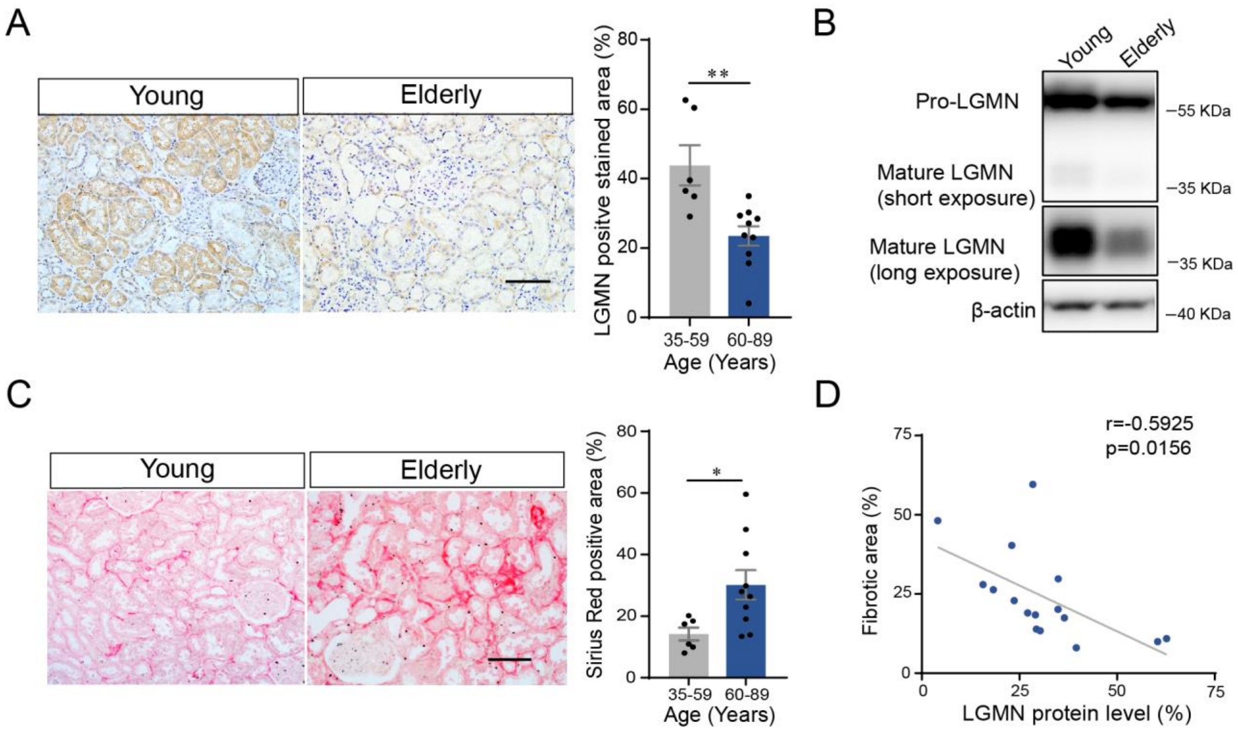

Supplemental Figure 2. Aging-related loss of LGMN correlates positively with renal fibrosis in human. Differently aged (35 to 86 years old, n = 16) kidney samples were obtained from patients of renal carcinoma underwent nephrectomy. Normal paracancerous sections were used for further analyzes. To analyze changes of LGMN expression during aging, IHC staining assay was performed. (A) Representative images and quantitative analysis of IHC staining of LGMN in young and elderly kidneys. Scale bar, 50 μm. (B) Representative images of mature LGMN in the whole kidney lysate assessed via western blotting. (C) Representative images and quantitative analysis of Sirius red staining in the kidney samples. Scale bar, 50 μm. (D) Correlation analysis of LGMN expression and Fibrosis. Data are presented as mean ± S.E.M. ***$p < 0.001$ **$p < 0.01$, *$p < 0.05$.

Supplementary Table 1. Personal Information of Human Samples

No.	Sex	Age (years old)	No.	Sex	Age (years old)
1	Male	57	10	Female	44
2	Male	63	11	Female	53
3	Male	63	12	Female	69
4	Male	66	13	Female	70
5	Male	73	14	Female	86
6	Male	81	15	Female	73
7	Male	71	16	Female	35
8	Male	46			
9	Male	35			

Supplementary Table 2. Primers for Quantitative PCR

Gene (homo-)	Forward (5'-3')	Reverse (5'-3')	Gene (mus-)	Forward (5'-3')	Reverse (5'-3')
$p16^{INK4\alpha}$	CCCAACGCACCGAATAGTTA	CCAGCGTGTCCAGGAAGCCCT	α-Sma	TCAGGGAGTAATGGTTGGAATG	GGTGATGATGCCGTGTTCTA
$p21^{CIP1}$	GTCACTGTCTTGTACCCTTGTG	GGCGTTTGGAGTGGTAGAAA	Fibronectin	GACTCATGGTGGCCACTAAATA	
IL6	CCCAGGAGAAGATTCCAAAGAT	GCTGCTTTCACACATGTTACTC	Collagen I	AGACCTGTGTGTTCCCTACT	GAATCCATCGGTCATGCTCTC
IL1α	CTGAAGGAGATGCCTGAGATAC	AAGCACACCCAGTAGTCTTG	$p16^{Ink4\alpha}$	GCTGGGTGGTCTTTGTGTA	TTAGCTCTGCTCTTGGGATTG
IL1β	ATGGACAAGCTGAGGAAGATG	CCCATGTGTCGAAGAAGATAGG	$p21^{Cip1}$	TTGTCGCTGTCTTGCACTC	GATAGAAATCTGTCAGGCTGGTC
MMP2	GGCACCCATTTACACCTACA	CCAAGGTCAATGTCAGGAGAG	il6	CTTCCATCCAGTTGCCTTCT	CTCCGACTTGTGAAGTGGTATAG
MMP9	GAACTTTGACAGCGACAAGAAG	CGGCACTGAGGAATGATCTAA	il10	TTGAATTCCCTGGGTGAGAAG	TCCACTGCCTTGCTCTTATTT
TGFβ1	CGTGGAGCTGTACCAGAAATAC	CACAACTCCGGTGACATCAA	il1α	CCTTACACCTACCAGAGTGATTT	AACCAAGTGGTGCTGAGATAG
PAI1	CTGGTGAATGCCCTCTACTTC	GGCGTGGTGAACTCAGTATAG	Cxcl10	TTTCTGCCTCATCCTGCTG	TCCCTATGGCCCTCATTCT
CTGF	GCCCAGACCCAACTATGATTAG	GGAGGCGTTGTCATTGGTAA	Cxcl12	CTCTGCATCAGTGACGGTAAA	CACAGTTTGGAGTGTTGAGGA
LGMN	GGACGTGGAAGATCTGACTAAA	CTGGCTTTGCGTTTCATACC	Mmp2	GTTCAACGGTCGGGAATACA	GCCATACTTGCCATCCTTCT
CXCL10	GGTGAGAAGAGATGTCTGAATCC	GTCCATCCTTGGAAGCACTGCA	Mmp9	CTGGAACTCACACGACATCTT	TCCACCTTGTTCACCTCATTT
CXCL12	CTCAACACTCCAAACTGTGCCC	CTCCAGGTACTCCTGAATCCAC	Vcam1	ACCTGCTCAAGTGATGGG	ATAAATGCCGGAATCGTC
VCAM1	GATTCTGTGCCCACAGTAAGGC	TGGTCACAGAGCCACCTTCTTG	Tgf-β1	CGAAGCGGACTACTATGCTAAA	TCCCGAATGTCTGACGTATTG
TFEB	AGTACCTGTCCGAGACCTATG	GTGTTGGGCATCTGCATTTC	Pai1	CTCCACAGCCTTTGTCATCT	ATTGTCTCTGTCGGGTTGTG
CTSD	GCAAACTGCTGGACATCGCTTG	GCCATAGTGGATGTCAAACGAGG	Ctgf	ACTATGATGCGAGCCAACTG	CTCCAGTCTGCAGAAGGTATTG
CTSB	GCTTCGATGCACGGGAACAATG	CATTGGTGTGGATGCAGATCCG	Lgmn	AGAGGATGTGACTCCAGAGAA	CCGTGGTCGGTGAAGTAAAT
			TFEB	GTCTGAGACCTATGGGAACAAG	TGCATCTCAGGGTTGATGTAG
			CtsD	TAAGACCACGGAGCCAGTGTCA	CCACAGGTTAGAGGAGCCAGTA
			CtsB	AGTCAACGTGGAGGTGTCTGCT	GTAGACTCCACCTGAAACCAGG
			Ulk1	GCAGCAAAGACTCCTGTGACAC	CCACTACACAGCAGGCTATCAG
			Vps34	GCTTCAGCCAAGCCTTGCTCAA	ACCGCCTTCATCAGATGGACCA
			Beclin	CAGCCTCTGAAACTGGACACGA	CTCTCCTGAGTTAGCCTCTTCC
			Bnip3	GCTCCAAGAGTTCTCACTGTGAC	GTTTTTCTCGCCAAAGCTGTGGC
			Atg12	GAAGGCTGTAGGAGACACTCCT	GGAAGGGGCAAAGGACTGATTC
			Atg5	CTTGCATCAAGTTCAGCTCTTCC	AAGTGAGCCTCAACCGCATCCT
			β-actin	CAGAAGGAGATTACTGCTCTGGCT	TACTCCTGCTTGCTGATCCACATC
